# Repositioning organohalogen drugs: a case study for identification of potent B-Raf V600E inhibitors via docking and bioassay

**DOI:** 10.1038/srep31074

**Published:** 2016-08-09

**Authors:** Yisu Li, Binbin Guo, Zhijian Xu, Bo Li, Tingting Cai, Xinben Zhang, Yuqi Yu, Heyao Wang, Jiye Shi, Weiliang Zhu

**Affiliations:** 1 CAS Key Laboratory of Receptor Research, Drug Discovery and Design Center, Shanghai Institute of Materia Medica, Chinese Academy of Sciences, Shanghai, 201203, China; 2 Nano Science and Technology Institute, University of Science and Technology of China, Suzhou, Jiangsu, 215123, China; 3 State Key Laboratory of Drug Research, Shanghai Institute of Materia Medica, Chinese Academy of Sciences, Shanghai, 201203, China; 4 UCB Biopharma SPRL, Chemin du Foriest, Braine-l’Alleud, Belgium

## Abstract

Drug repositioning has been attracting increasingly attention for its advantages of reducing costs and risks. Statistics showed that around one quarter of the marketed drugs are organohalogens. However, no study has been reported, to the best of our knowledge, to aim at efficiently repositioning organohalogen drugs, which may be attributed to the lack of accurate halogen bonding scoring function. Here, we present a study to show that two organohalogen drugs were successfully repositioned as potent B-Raf V600E inhibitors *via* molecular docking with halogen bonding scoring function, namely D^3^DOCKxb developed in our lab, and bioassay. After virtual screening by D^3^DOCKxb against the database CMC (Comprehensive Medicinal Chemistry), 3 organohalogen drugs that were predicted to form strong halogen bonding with B-Raf V600E were purchased and tested with ELISA-based assay. In the end, 2 of them, rafoxanide and closantel, were identified as potent inhibitors with IC_50_ values of 0.07 μM and 1.90 μM, respectively, which are comparable to that of vemurafenib (IC_50_: 0.17 μM), a marketed drug targeting B-Raf V600E. Single point mutagenesis experiments confirmed the conformations predicted by D^3^DOCKxb. And comparison experiment revealed that halogen bonding scoring function is essential for repositioning those drugs with heavy halogen atoms in their molecular structures.

Drug repositioning is getting progressively attention as a promising method for drug discovery. A repositioned compound with proven bioavailability and known safety profiles has a lot of advantages such as an accelerated R&D process, reduced development cost, and decreased failure rate due to safety^1^. Impressively, with the growing computing ability of computers, computational repositioning promotes the advantages of drug repositioning to a new level^2^,^3^.

Many systematic computational repositioning strategies have been published and molecular docking is a vital methodology among them, which is also known as structure-based virtual screening^2^,^4^,^5^,^6^,^7^. Molecular docking was pioneered during the early 1980s, and remains a highly active area of research until now^8^. It allows the rapid and cost-effective evaluation of the interactions between large libraries of compounds and biomolecular targets. With the help of molecular docking, new drug candidates could be developed faster with lower cost^9^,^10^.

There have been numerous drug repositioning studies based on molecular docking over the last decade^11^. Huang and co-workers utilized molecular docking to identify new 5-HT_2A_ inhibitors. In their study, a well-known multiple kinase inhibitor sorafenib showed unexpected 5-HTRs binding affinities in molecular docking, which was verified in the following experimental study^12^. Bisson *et al.* identified androgen receptor (AR) antagonists from a database of existing drugs by using molecular docking, which three marketed antipsychotic drugs were found to exhibit anti-AR transactivation efficacies experimentally^13^. Chan *et al.* performed virtual screening on an FDA-approved drug database of over 3,000 compounds. A compound identified by virtual screening was found to stabilize the c-myc Pu27 G-quadruplex in a dose-dependent fashion^14^.

In spite of all these successes and evolving computers, the rate of yielding successful repositioning drugs from molecular docking remains unsatisfied. This phenomenon can be attributed to various reasons, among which the accuracy of scoring functions for docking is definitely a key factor. Scoring function which ranks the poses generated by docking software directly decides the final docking conformations of the compounds and its priority. Therefore, the accuracy of scoring function influences the results of molecular docking to a great extent^9^,^15^. However, current scoring functions are imperfect, especially, in dealing with halogen bonding which is dominated by the noncovalent attractive interaction between the σ-hole of drugs’ halogen atoms and a nucleophile in target proteins^16^,^17^,^18^. As around 25% drugs are organohalogens, halogen bonding is playing an increasingly important role in drug discovery^19^,^20^,^21^,^22^,^23^. Consequently, the imperfection in dealing with halogen bonding influences the accuracy of scoring function to a great extent for drug repositioning as well. There are several docking scoring functions emerged to fill the gaps in this area^24^,^25^,^26^. Recently, our laboratory developed a docking software, namely D^3^DOCKxb, which showed good performance in a docking power evaluation among test sets with halogen bonding interactions due to its reliable halogen bond scoring function^27^,^28^. To the best of our knowledge, there is no report on drug repositioning by taking into account halogen bonding interactions. Therefore, we attempted to apply D^3^DOCKxb on the repositioning of organohalogen drugs.

B-Raf is an extensively investigated serine/threonine kinase which is a member of the RAS/RAF/MEK/ERK pathway. The B-Raf protein kinase is mutated in a broad range of human cancers and especially in malignant melanoma with the highest incidence of 60–70%, and it is considered as a promising therapeutic target^29^. B-Raf V600E mutation is dominant which occurs in more than 90% malignant melanoma with B-Raf mutations. Marketed drugs like vemurafenib and dabrafenib have been developed. However, the drug resistance problem^30^,^31^ of those inhibitors generated imperative needs for novel B-Raf V600E inhibitors.

In this study, we performed virtual screening using D^3^DOCKxb on drugs with heavy halogen atoms (Cl, Br, and I) from CMC (Comprehensive Medicinal Chemistry) to investigate the role of halogen bonding in drug repositioning. The selected organohalogen drugs with predicted halogen bonding patterns by D^3^DOCKxb were tested by bioassay. We discovered two potent B-Raf V600E organohalogen inhibitors from the marketed drugs, and the halogen bonding patterns were confirmed by single point mutagenesis experiments. Moreover, the comparison between docking conformations of the two inhibitors by different software demonstrated the superiority of D^3^DOCKxb in predicting halogen bonding. In conclusion, for the first time, molecular docking with halogen bonding scoring function successfully repositioned two organohalogen drugs as potent B-Raf V600E inhibitors. Therefore, halogen bonding should be taken into account for improving success rate of organohalogen drug repositioning.

## Results

### Structure-based virtual screening and compound selecting

**Figure 1:**
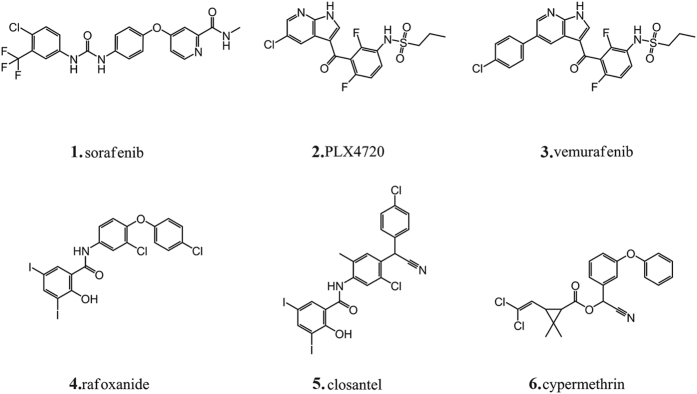
Molecular structures of the repositioned old drugs and the positive drugs in virtual screening and bioassay.

The virtual screening against B-Raf (PDB IDs: 1UWJ and 3C4C) was carried out on 1,634 organohalogen drugs by D^3^DOCKxb using default parameters. The threshold values of the docking score for 1UWJ and 3C4C are −11.42 Kcal/mol (sorafenib) and −10.40 Kcal/mol (PLX4720) respectively (Table 1), which acquired by re-docking the positive drugs into its crystal structure. 67 organohalogen drugs with docking scores better than the positive controls and docking conformations with halogen bonding interactions were selected for further evaluation. After careful visual inspection, 3 drugs, namely rafoxanide, closantel and cypermethrin (Fig. 1), were selected and purchased for further experimental assays (Table 1).

### B-Raf V600E inhibitory activities

**Figure 2:**
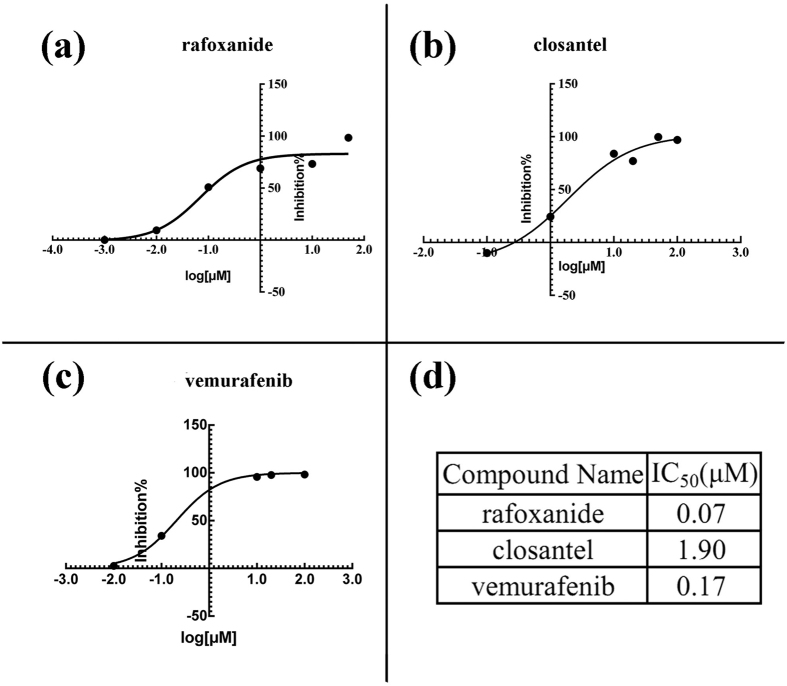
Inhibitory activities of the compounds against B-Raf V600E. The fitted IC_50_ curves of rafoxanide (**a**), closantel (**b**) and vemurafenib (**c**). IC_50_ value of the three compounds are listed in (**d**).

ELISA-based assay showed that rafoxanide and closantel possess potent activity against B-Raf V600E with inhibitory rates of 73.2% and 83.9% at 10 μM, respectively, while cypermethrin was inactive (Table 1). Thus, the IC_50_ values were determined for rafoxanide and closantel to be 0.07 μM and 1.90 μM, respectively, which is appreciable compared to positive control vemurafenib (IC_50_ = 0.17 μM, Fig. 2).

### Halogen bonding of rafoxanide and closantel with B-Raf V600E

**Figure 3:**
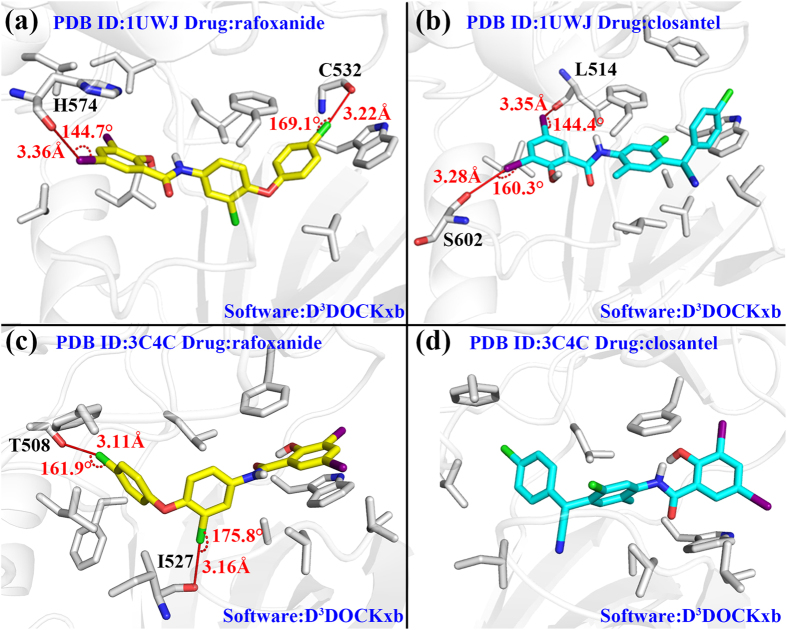
The binding modes of rafoxanide and closantel docked in 1UWJ and 3C4C. (**a**) rafoxanide docked in 1UWJ with D^3^DOCKxb. (**b**) closantel docked in 1UWJ with D^3^DOCKxb. (**c**) rafoxanide docked in 3C4C with D^3^DOCKxb. (**d**) closantel docked in 3C4C with D^3^DOCKxb. The yellow compound stands for rafoxanide and the cyan compound stands for closantel. Halogen bonds were labeled with distance and angles. The surrounding protein residues interact with the compounds are presented in gray stick model.

The best scored docking conformations in the top clusters of rafoxanide and closantel were shown in Fig. 3, and the geometrical parameters of the predicted halogen bonding were summarized in Table 2. Figure 3a illustrates the docking mode of rafoxanide to 1UWJ. Two halogen bonds are formed at each end of the compound via Cl and I atoms. Figure 3b shows the binding mode of closantel to 1UWJ. Two potent I-O type halogen bonds are formed with Leu514 and Ser602, respectively. In terms of position in the binding pocket, rafoxanide, compared to closantel, buried deeper into the binding pocket. This could be attributed to the cyan group which impeded the entry of closantel by steric hindrance.

Figure 3c,d display the docking results against 3C4C. Different from the results against 1UWJ, rafoxanide and closantel exhibited a reversed docking conformation in the binding pocket. This is understandable since 1UWJ is DFG-out inactive conformation while 3C4C is DFG-in active conformation binding with sorafenib and PLX4720 respectively. Two Cl-O type halogen bonds with typical geometry parameters were discovered on the binding mode between rafoxanide and 3C4C (Fig. 3c). No halogen bonding interactions were found according to the docking results between closantel and 3C4C (Fig. 3d).

Furthermore, the top10 conformations were presented in each case for a more comprehensive analyzation. Supplementary Table S1 showed the docking scores of the top10 conformations and its cluster information. Supplementary Fig. S1 illustrated all the top10 conformations according to their clusters in the binding pocket.

For rafoxanide docked in 1UWJ, the top10 conformations came from a single cluster and showed a great consistency which made the best scored conformation the most reliable one. The same situation happened in closantel docked in 1UWJ and rafoxanide docked in 3C4C. For closantel docked in 3C4C, two clusters were observed. However, cluster1(contains the best scored conformation) conformations had better consistency and scores, which also made the best scored conformation the most reliable one for analyzation.

### Single point mutagenesis experiments

Ser602 and Thr508, whose sidechains formed halogen bonds with rafoxanide and closantel, were mutated to alanine. Table 3 and Supplementary Fig. S2 showed the IC_50_ value of rafoxanide and closantel against the mutated and wild type B-Raf.

The positive drug vemurafenib inhibited mutated and wild type B-Raf with the same IC_50_ level (0.11 ~ 0.39 μM), which showed its equally potency in those 4 cases. Both rafoxanide and closantel showed high potency against the wild type B-Raf and B-Raf V600E (0.07 ~ 1.94 μM). In the cases that halogen bonding acceptor residues were mutated (B-Raf T508A and B-Raf S602A), however, rafoxanide and closantel showed significantly decreased potency with an increased IC_50_ value (Table 3). These results clearly demonstrated that Ser602 and Thr508 played an important role (halogen bonding) in the binding mode of rafoxanide and closantel, since the positive drug vemurafenib was uninfluenced in the above 4 cases (no halogen bonding interactions with the binding pocket according to D^3^DOCKxb).

### Comparison with other docking software

Before we carried out the comparison, the positive drugs (sorafenib for 1UWJ, PLX4720 for 3C4C) were re-docked in their crystal structures to verify the docking ability of the three docking software in those two structures. Top20 conformations were presented in Supplementary Fig. S3 and scores and rmsd to the crystal structures were listed in Supplementary Table S2. The top20 docking conformations of the positive drugs predicted from D^3^DOCKxb and Autodock had little difference in each case. All of the conformations had relatively low rmsd values to the crystal structure (Supplementary Fig. S3a,b,d,e). There was an exception in Supplementary Fig. S3d where the best scored conformation deviated from the crystal structure to a great extent. However, due to the strong and stable performance of the rest conformations we still thought that D^3^DOCKxb achieved a successful docking. In the case of Glide, despite the inconsistency of the top20 conformations (Supplementary Fig. S3c,f), the low rmsd values of the top conformations (<1 Å) demonstrated the capacity of Glide in successfully docking in 1UWJ and 3C4C (Supplementary Table S2). In conclusion, all three docking software performed well in reproducing the crystal structure of positive drugs in 1UWJ and 3C4C.

**Figure 4:**
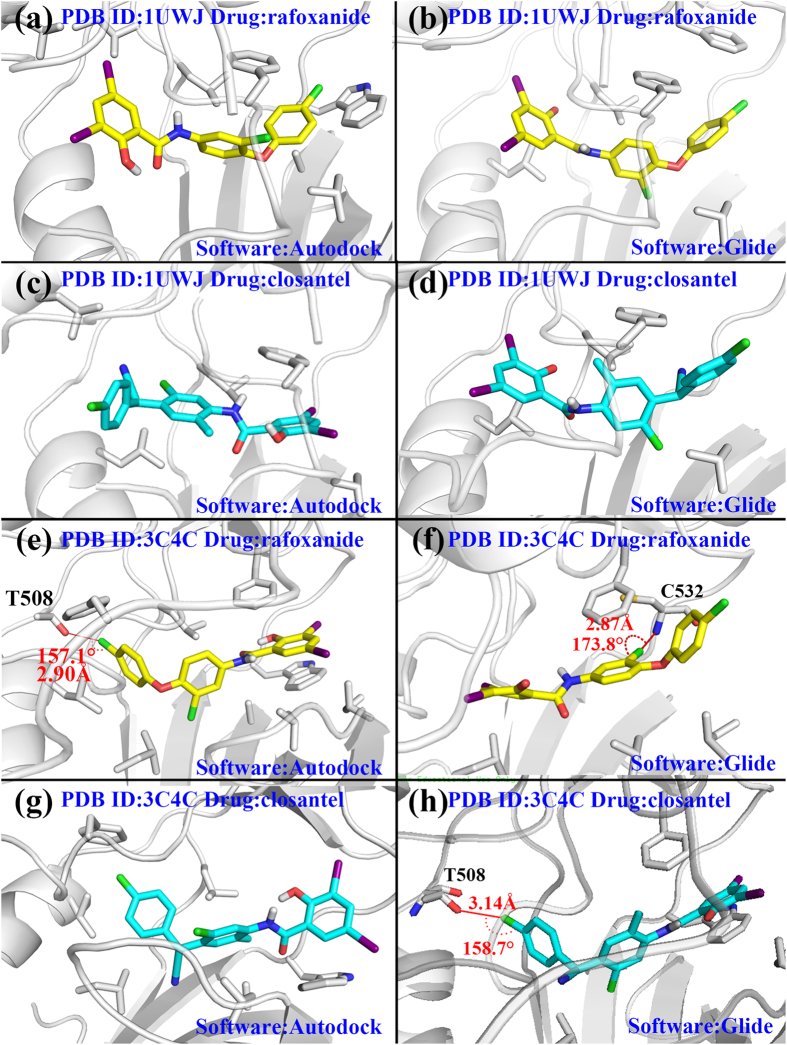
The binding modes from Autodock and Glide. (**a**) rafoxanide docked in 1UWJ with Autodock. (**b**)rafoxanide docked in 1UWJ with Glide. (**c**) closantel docked in 1UWJ with Autodock. (**d**) closantel docked in 1UWJ with Glide. (**e**) rafoxanide docked in 3C4C with Autodock. (**f**) rafoxanide docked in 3C4C with Glide. (**g**) closantel docked in 3C4C with Autodock. (**h**) closantel docked in 3C4C with Glide. The yellow compound stands for rafoxanide and the cyan compound stands for closantel. Halogen bonds were labeled with distance and angles. The surrounding protein residues interact with the compounds are presented in gray stick model.

We re-docked rafoxanide and closantel by Autodock and Glide (D^3^DOCKxb conformations already existed), and for each case, top10 docking conformations were extracted and clustered for further analyzation. Supplementary Figs S4 and S5 illustrated clusters in different cases, and Supplementary Tables S3 and S4 demonstrated the binding scores and rmsd values towards the best scored conformation in each cluster. All in all, the best scored conformations usually belonged to the largest clusters in each case which made them the most reliable choice for further analyzation. And in cases with multiple clusters(rafoxanide docked in 1UWJ by Autodock, closantel docked in 1UWJ by Glide), cluster1 conformations(contains the best scored conformation) had better interactions with the binding pocket. Therefore, we used the best scored conformation in each case to compare the performance of three different software. All the relevant information about the comparison were listed in Table 2 and Fig. 4 shows the binding modes from Autodock and Glide as a complement to Fig. 3.

For rafoxanide docking in 1UWJ, two halogen bonds were predicted by the D^3^DOCKxb software (Fig. 3a). The 3-chlorophenyl group at one end of rafoxanide forms a typical halogen bond with amide oxygen atom from Cys532 with *d* = 3.22 Å and *θ* = 169.1°. An iodine atom from the 2-hydroxy-3,5-diiodobenzamid group at the other end of the compound formed a I-O type halogen bond with His574 with *d* = 3.36 Å and *θ* = 144.7°. While Autodock and Glide generated conformations which resembled the D^3^DOCKxb’s conformation to a great extent, their conformations failed to form any halogen bonding interactions due to the small difference from the D^3^DOCKxb’s conformation (Fig. 4a,b).

For closantel docking in 1UWJ, three docking software produced three largely different conformations. Two potent I-O type halogen bonds were predicted by D^3^DOCKxb between the 2-hydroxy-3,5-diiodobenzamid group of closantel and the residues of the protein (Leu514 and Ser602). The conformation predicted by AutoDock was approximately a reversed version of the D^3^DOCKxb conformation, and no halogen bond was formed in this case (Fig. 4c). As for the results from Glide, the difference from D^3^DOCKxb’s conformation caused no halogen bonding in the 2-hydroxy-3,5-diiodobenzamid group (Fig. 4d).

For rafoxanide docked in 3C4C by D^3^DOCKxb, two typical Cl-O type halogen bonds were formed. Although the conformation generated by AutoDock resembled the D^3^DOCKxb results to a great extent, it only predicted the halogen bonding in the chlorophenyl group of the compound. The halogen bonding in the chlorophenoxy group from D^3^DOCKxb was not predicted by AutoDock (Fig. 4e). On the other hand, the results from Glide differs the above two conformations to a large scale. Instead of forming halogen bond with I527, the chloro benzene groups in the middle of the compound formed halogen bond with C532 (Fig. 4f).

For closantel docking in 3C4C, the conformations from D^3^DOCKxb and Autodock resembled each other to a great extent (Figs 3d and 4g). However, neither of them predicted any halogen bonding in this case. The results from Glide predicted an Cl-O type halogen bond in this case (Fig. 4h).

## Discussion

In general, D^3^DOCKxb clearly shows its power in predicting halogen bonding in the aforementioned cases with 6 halogen bonds in total while AutoDock only predicted 1 halogen bonds and Glide only predicted 2. Despite the conformational similarities, the D^3^DOCKxb are more accurate in predicting halogen bonding than AutoDock and Glide. The docking scores from D^3^DOCKxb, Glide and Autodock are listed in Table 4. All three docking software gave relatively better docking scores to positive drugs of 1UWJ and 3C4C, and we already proved the reliability of docking positive drugs by conformational analysis above. We can observe that D^3^DOCKxb gave rafoxanide and closantel comparatively high evaluations, and the total binding scores by D^3^DOCKxb are more consistent with the bioassay results than that by other software. For instance, D^3^DOCKxb predicted stronger binding between rafoxanide and B-Raf V600E than that of closantel by about 2 kcal/mol, while both AutoDock and Glide predicted very similar binding strength between the two drugs and B-Raf V600E. In fact, rafoxanide is 27 times active than closantel in terms of IC_50_ value (0.07 μM vs 1.90 μM). Single point mutagenesis experiments further validated the importance of the halogen bonds we predicted by D^3^DOCKxb, therefore, confirmed the conformations predicted by D^3^DOCKxb from an experimental point of view.

In conclusion, this study performed virtual screening against organohalogen drugs in CMC database using D^3^DOCKxb, a docking software that could deal with halogen bonding accurately, for repositioning the drugs as B-Raf inhibitor. Based on the docking result, 3 organohalogen drugs were purchased for experimental study. The bioassay results revealed that two organohalogen drugs, namely, rafoxanide and closantel, were potent inhibitors of B-Raf V600E inhibitors with IC_50_ values comparable to that of the marketed drug vemurafenib. On the other hand, we used single point mutagenesis experiments to verify the halogen bonding patterns predicted by D^3^DOCKxb. Furthermore, this result demonstrated that docking software with halogen bonding scoring function is essential, especially, in the research of repositioning organohalogen drugs.

## Methods

### Ligands and proteins

The CMC database (version 2011.2) collects drug molecules from 1900 until 2010. Among the 9,099 drug molecules in the database, 1,634 drug compounds contained Cl, Br and I atoms, which are known as heavy halogen atoms, were selected for the repositioning study of organohalogen drugs via docking approach. In other words, 18% of the drugs from CMC database have the possibility to form halogen bonding with pharmaceutical targets, which shows the prevalence and importance of halogen bonding in pharmaceutical research. The acquired organohalogen drug molecules were then prepared by the LigPrep module (version 2.4, Schrödinger, LLC, New York, NY, 2010) at the pH value of 7 using Epik (version 2.1, Schrödinger, LLC, New York, NY, 2010) for the ionization state generation^32^,^33^.

The B-Raf structures used in this study were downloaded from Protein Data Bank (PDB) with PDB IDs 1UWJ^34^ and 3C4C^35^. 1UWJ is DFG-out inactive conformation and 3C4C is DFG-in active conformation of the kinase. Protein Preparation Wizard in maestro (version 9.1, Schrödinger, LLC, New York, NY, 2010) was used to prepare the protein structures for virtual screening^36^.

### Virtual screening by D^3^DOCKxb

The virtual screening procedure was performed by D^3^DOCKxb, which was developed for including the effects of halogen bonding in drug discovery by seamlessly incorporating two different halogen bonding scoring functions into AutoDock (version 4.2)^37^,^38^. One is a quantum mechanics-based scoring function for halogen bonding interaction namely XBScore^QM^ which showed strong ability to predict halogen bonding^27^. The other is a knowledge-based halogen bond scoring function, termed XBPMF, developed by an iterative method^28^. In this study, we performed virtual screening using XBScore^QM^ scoring function due to its verified good performance in describing halogen bonding^27^. Since the only difference between AutoDock and D^3^DOCKxb lies in their scoring functions, all the calculation parameters used for D^3^DOCKxb in this study came from the default parameters in AutoDock.

### Compound selecting

**Figure 5:**
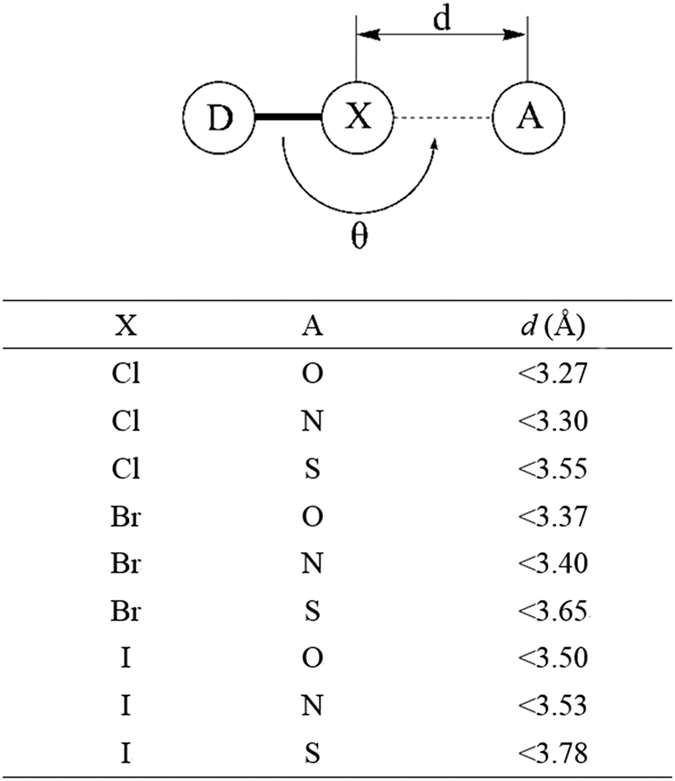
Geometrical definition of halogen bonds in this study. The model on the top shows a typical halogen bond model where halogen atoms (**X**) shared by donor (**D**) and acceptor (**A**) When X and A stands for different atoms, the corresponding distance restrictions are listed on the table below. *θ* is the angle of the halogen bond which is required to be larger than 140°.

To identify potential inhibitors from the results of virtual screening, two steps were applied. The first step was to set a threshold value of the docking scores by re-docking the crystallized ligands (sorafenib in 1UWJ and PLX4720 in 3C4C) as positive controls, which means we only focused on the drugs with docking scores better than the value of the positive controls. The second step was to identify potential halogen bonding interactions between the organohalogen drugs and B-Raf V600E predicted by the docking. And the criteria for halogen bonding interactions patterns in this study were defined as: the distance (*d*) between halogen atoms and acceptors is less than the sum of their van der Waals radii, and the bond angle (*θ*) is larger than 140° because halogen bonding is highly directional (Fig. 5)^16^,^19^,^22^,^23^,^39^.

### Experimental assay

An ELISA-based assay was performed to assay the inhibitory activities of the purchased organohalogen drugs against B-Raf V600E. In this assay, compounds of different concentrations impaired the catalytic activity of B-Raf V600E which converts MEK protein to p-MEK protein, and the p-MEK can be detected by immunoblotting assay. Vemurafenib was used as positive control.

### Conformation analyzation

The compounds were re-docked with the ga_run parameter value of 100 in order to achieve a more precise docking results. Top10 conformations were extracted and analyzed to prevent the case that the real conformation was not the best scored one. Halogen bonds and other interactions were identified according to the definition.

### Single point mutagenesis experiments

The mutant sequence of human BRAF kinase domain (B-RAFT508A and B-RAFS602A, residues 433–726) was inserted into the plasmid of pFastBacTM Dual. Then the recombinant plasmid was transported into the sf9 cells. Mutant protein was expressed and purified respectively.

### Docking comparison

D^3^DOCKxb was compared to AutoDock (version 4.2) and Glide (Glide, version 5.6, Schrödinger, LLC, New York, NY, 2010)^40^,^41^,^42^ to evaluate its ability to identify halogen bonding interactions. During the docking process of D^3^DOCKxb and AutoDock, Ga_run parameter was set to 100. For the Glide cases, we employed the SP mode. The docked conformations were visualized with PyMOL (The PyMOL Molecular Graphics System, version 1.3 Schrödinger, LLC.) for further analyzation.

## Additional Information

**How to cite this article**: Li, Y. *et al.* Repositioning organohalogen drugs: a case study for identification of potent B-Raf V600E inhibitors via docking and bioassay. *Sci. Rep.* **6**, 31074; doi: 10.1038/srep31074 (2016).

## Supplementary Material

Supplementary Information

## Figures and Tables

**Table 1:** Docking scores from D^3^DOCKxb, and inhibitory activities against B-Raf V600E.

Compound Name	Docking Score (1UWJ)	Docking Score (3C4C)	Inhibition Rate (at 10 μM)
rafoxanide	−14.02	−12.70	73.20%
closantel	−12.08	−11.15	83.90%
cypermethrin	−11.95	−11.46	17.60%
sorafenib	−11.42	−10.13	96.99%
PLX4720	−10.39	−10.40	82.50%
vemurafenib	−12.32	−10.93	82.90%

**Table 2:** Detailed information about the docking results from three different docking software.

PDB ID	Compound Name	Docking Software	Halogen Bond Number	Halogen Bond Donors	Halogen Bond Acceptors	Halogen Bond Geometrical Parameters (Å/°)
1UWJ	rafoxanide	D^3^DOCKxb	2	I	H574	3.36/144.7
				Cl	C532	3.22/169.1
		AutoDock	0	—	—	—
		Glide	0	—	—	—
	closantel	D^3^DOCKxb	2	I	S602	3.28/160.3
				I	L514	3.35/144.4
		AutoDock	0	—	—	—
		Glide	0	—	—	—
3C4C	rafoxanide	D^3^DOCKxb	2	Cl	T508	3.11/161.9
				Cl	I527	3.16/175.8
		AutoDock	1	Cl	T508	2.90/157.1
		Glide	1	I	C532	2.87/173.8
	closantel	D^3^DOCKxb	0	—	—	—
		AutoDock	0	—	—	—
		Glide	1	I	A481	3.14/158.7

Detailed geometrical parameters were given for halogen bonding.

**Table 3:** The IC_50_ value of rafoxanide, closantel and vemurafenib towards wild type B-Raf and 3 mutated B-Raf.

Compound	B-RAF^WT^	B-RAF^V600E^	B-RAF^T508A^	B-RAF^S602A^
	IC_50_(μM)	IC_50_(μM)	IC_50_(μM)	IC_50_(μM)
rafoxanide	1.94	0.07	15.70	11.41
closantel	1.89	1.90	5.97	6.35
vemurafenib	0.31	0.17	0.11	0.39

**Table 4:** Total binding scores from D^3^DOCKxb, Glide and Autodock are listed in column 3–5.

PDB ID	Compound Name	D^3^DOCKxb	Autodock	Glide
1UWJ	**sorafenib**	**−11.42**	**−11.18**	**−12.08**
	PLX4720	**−**10.39	**−**10.27	−11.24
	rafoxanide	**−**14.02	**−**12.00	−8.61
	closantel	**−**12.08	**−**12.53	−8.73
3C4C	**PLX4720**	**−10.40**	**−10.20**	**−11.45**
	sorafenib	**−**10.13	**−**10.08	−6.28
	rafoxanide	**−**12.70	**−**10.72	−7.21
	closantel	**−**11.15	**−**11.25	−7.66

Positive drugs of 1UWJ and 3C4C are emphasized with bold.
