# Supplementary material for: Repositioning organohalogen drugs: a case study for identification of potent B-Raf V600E inhibitors via docking and bioassay
Source: Sci Rep. 2016 Aug 9;6:31074. doi: 10.1038/srep31074 (PMC4977465; doi:10.1038/srep31074)
Supplement: Supplementary Information [file srep31074-s1.pdf]

Supplementary Information

Repositioning organohalogen drugs: a case study for identification of potent B-Raf V600E inhibitors via docking and bioassay

Yisu Li, [†,§,#] Binbin Guo, [†,#] Zhijian Xu, [,†,⊥] Bo Li,[†] Tingting Cai, [†] Xinben Zhang, [†] Yuqi Yu, [†] Heyao Wang, [*,†] Jiye Shi, [‡] Weiliang Zhu [*,†]*

[†]CAS Key Laboratory of Receptor Research, Drug Discovery and Design Center, Shanghai Institute of Materia Medica, Chinese Academy of Sciences, Shanghai, 201203, China

[§]Nano Science and Technology Institute, University of Science and Technology of China, Suzhou, Jiangsu, 215123, China

[⊥]State Key Laboratory of Drug Research, Shanghai Institute of Materia Medica, Chinese Academy of Sciences, Shanghai, 201203, China

[‡]UCB Biopharma SPRL, Chemin du Foriest, Braine-l'Alleud, Belgium

*To whom correspondence should be addressed. Phone: +86-21-50806600-1201 (Z.X.), +86-21-50805785 (H.W.), +86-21-50805020 (W.Z.), Fax: +86-21-50807088 (W.Z.), E-mail: zjxu@simm.ac.cn (Z.X.), hywang@simm.ac.cn (H.W.), wlzhu@mail.shcnc.ac.cn (W.Z.).
#These authors contributed equally to the work.

Supplementary Table S1. Cluster information of rafoxanide and closantel docked in 1UWJ and 3C4C by D^3DOCKxb. Binding scores and the rmsd towards the best scored conformation in cluster were listed. (Page S3)

Supplementary Figure S1. Rafoxanide and closantel docked in 1UWJ and 3C4C by D^3DOCKxb. Top10 conformations were clustered in each case. Halogen bond interactions were labeled according to the best scored conformation in each cluster. (Page S4)

Supplementary Figure S2. The IC_{50} curves of vemurafenib, rafoxanide and closantel towards wild type B-Raf, B-Raf T508A and B-Raf S602A. (Page S5)

Supplementary Figure S3. D^3DOCKxb, Autodock and Glide re-docked the positive drugs in 1UWJ and 3C4C. Top20 conformations were extracted and demonstrated. Crystal structures were highlighted in red color. (Page S6)

Supplementary Table S2. Docking results of sorafenib and PLX4720 (positive drugs) in 1UWJ and 3C4C. Predicted binding scores from D^3DOCKxb, Autodock and Glide were listed and the rmsd towards the crystal structure were also listed. (Page S7)

Supplementary Figure S4. Rafoxanide and closantel docked in 1UWJ and 3C4C by Autodock. Top10 conformations were clustered in each case. Halogen bond interactions were labeled according to the best scored conformation in each cluster. (Page S8)

Supplementary Figure S5. Rafoxanide and closantel docked in 1UWJ and 3C4C by Glide. Top10 conformations were clustered in each case. Halogen bond interactions were labeled according to the best scored conformation in each cluster. (Page S9)

Supplementary Table S3. Cluster information of rafoxanide and closantel docked in 1UWJ and 3C4C by Autodock. Binding scores and the rmsd towards the best scored conformation in cluster were listed. (Page S10)

Supplementary Table S4. Cluster information of rafoxanide and closantel docked in 1UWJ and 3C4C by Glide. Binding scores and the rmsd towards the best scored conformation in cluster were listed. (Page S11)

Supplementary Table S1. Cluster information of rafoxanide and closantel docked in 1UWJ and 3C4C by D^3DOCKxb. Binding scores and the rmsd towards the best scored conformation in cluster were listed.

Software	PDB ID	Drug	Cluster	Binding Score Rank	Binding Score (kcal/mol)	Cluster RMSD(Å)
D^3DOCKxb	1UWJ	rafoxanide	1	1	-14.02	0.00
				2	-14.02	0.04
				3	-14.01	0.05
				4	-14.01	0.04
				5	-14.01	0.05
				6	-14.01	0.08
				7	-14.00	0.08
				8	-14.00	0.09
				9	-14.00	0.03
				10	-14.00	0.03
		closantel	1	1	-12.08	0.00
				2	-12.06	0.23
				3	-11.21	1.26
				4	-11.10	0.81
				5	-11.09	0.80
				6	-11.03	1.23
				7	-10.88	1.22
				8	-10.79	1.22
				9	-10.69	1.24
				10	-10.64	1.20
	3C4C	rafoxanide	1	1	-12.70	0.00
				2	-12.67	0.06
				3	-12.67	0.16
				4	-12.66	0.09
				5	-12.66	0.20
				6	-12.66	0.21
				7	-12.65	0.22
				8	-12.65	0.21
				9	-12.65	0.24
				10	-12.65	0.05
		closantel	1	1	-11.15	0.00
				2	-11.15	0.11
				3	-11.13	0.12
				4	-10.88	0.14
			2	1	-10.84	0.00
				2	-10.78	0.10
				3	-10.70	0.17
				4	-10.64	0.09
				5	-10.59	0.10
				6	-10.55	1.00

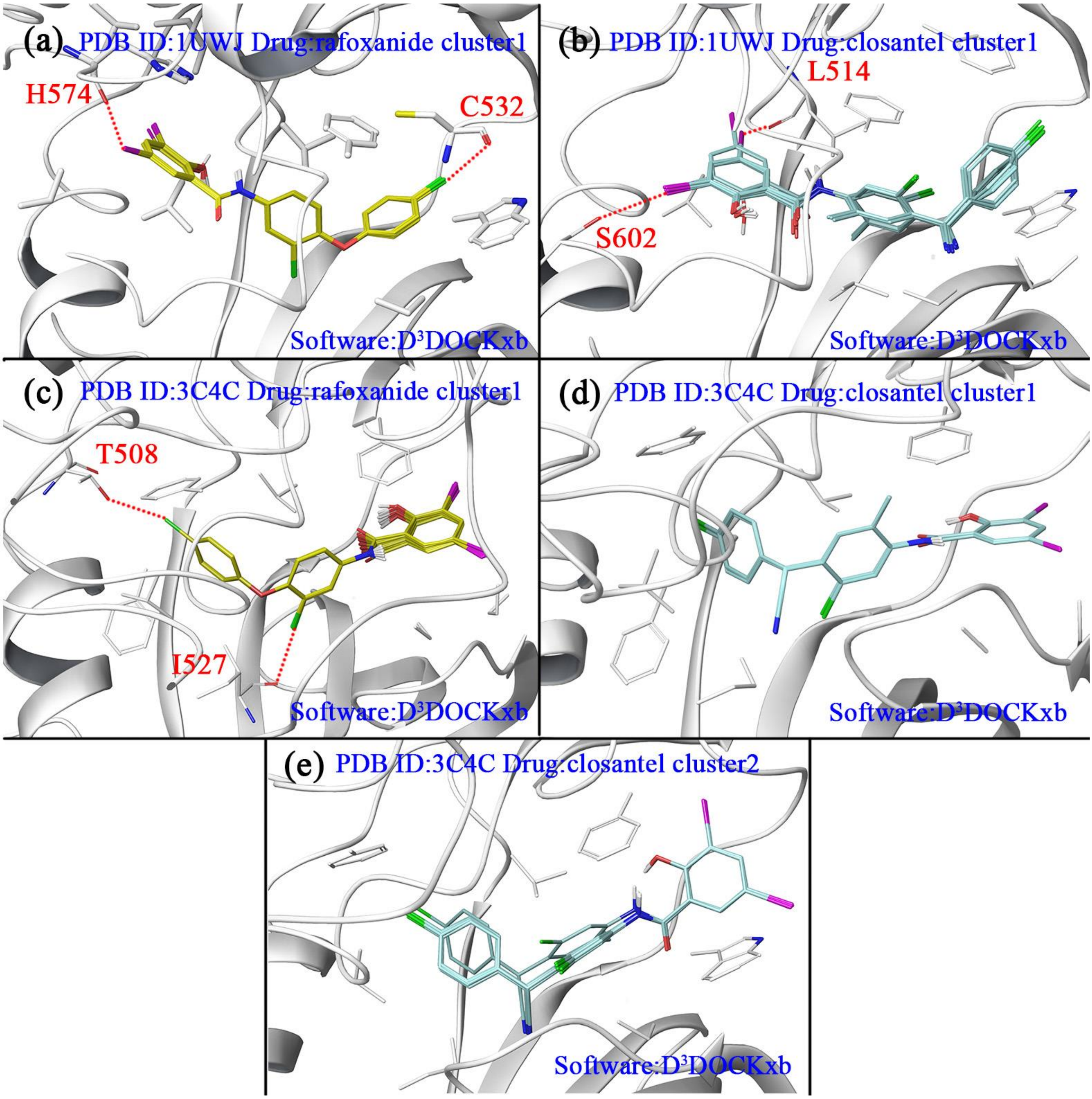

Supplementary Figure S1. Rafoxanide and closantel docked in 1UWJ and 3C4C by D^3DOCKxb. Top10 conformations were clustered in each case. Halogen bond interactions were labeled according to the best scored conformation in each cluster.

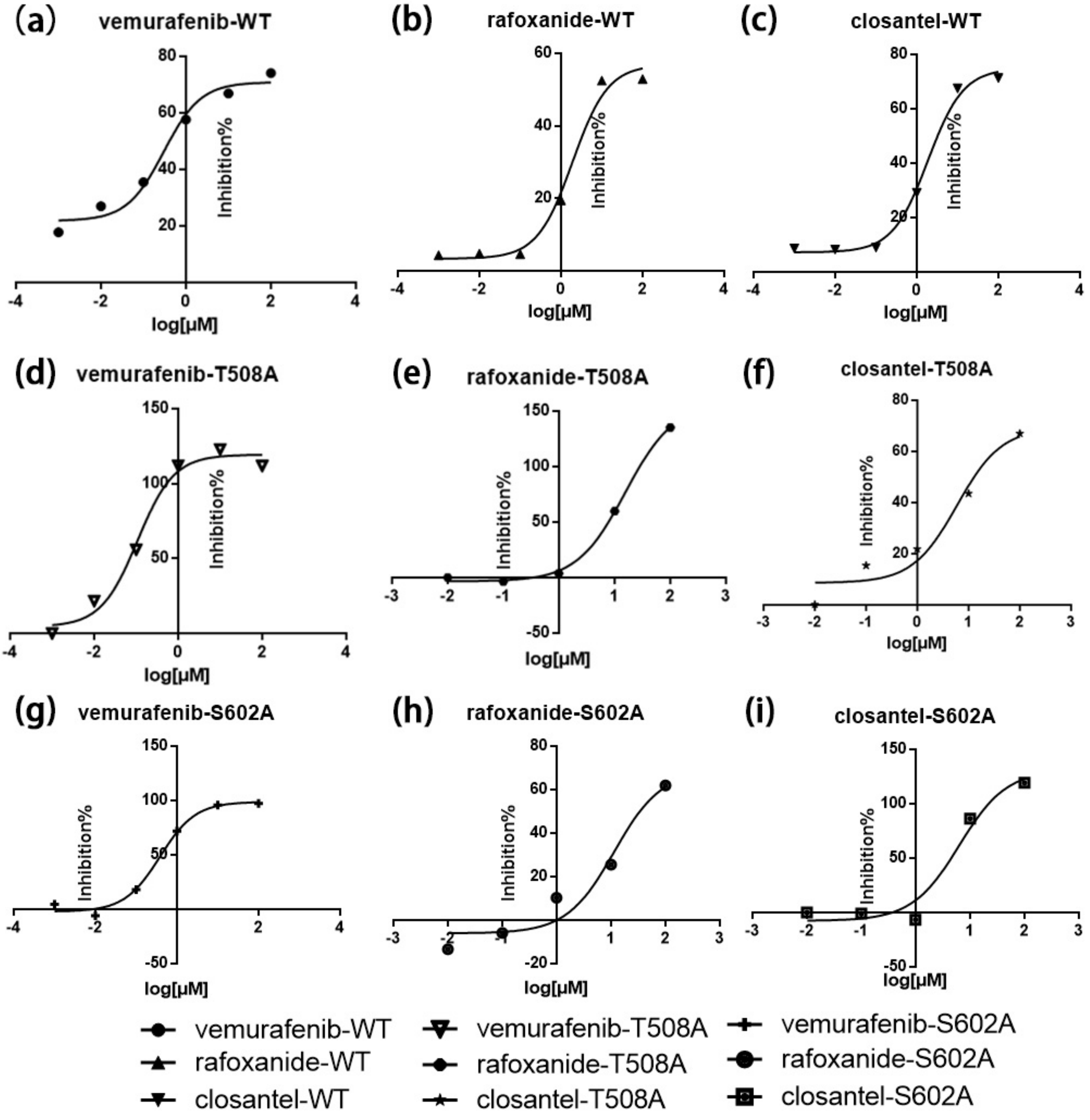

Supplementary Figure S2. The IC_{50} curves of vemurafenib, rafoxanide and closantel towards wild type B-Raf, B-Raf T508A and B-Raf S602A.

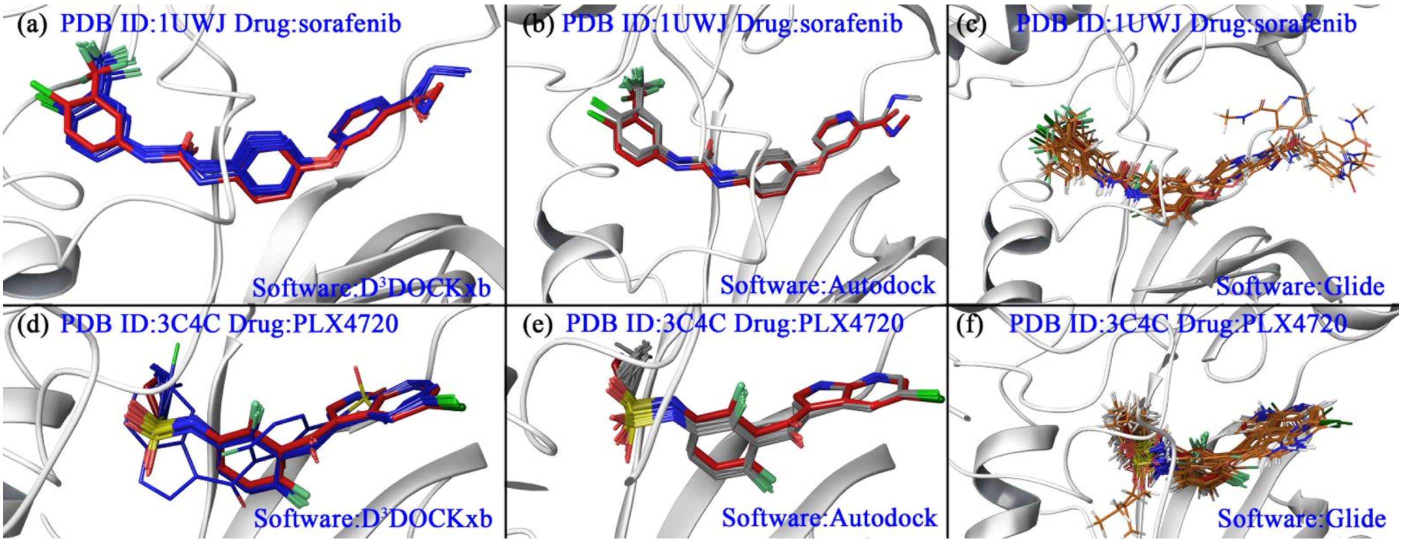

Supplementary Figure S3. D^{3}DOCKxb, Autodock and Glide re-docked the positive drugs in 1UWJ and 3C4C. Top20 conformations were extracted and demonstrated. Crystal structures were highlighted in red color.

Supplementary Table S2. Docking results of sorafenib and PLX4720 (positive drugs) in 1UWJ and 3C4C. Predicted binding scores from D^3DOCKxb, Autodock and Glide were listed and the rmsd towards the crystal structure were also listed.

Rank	Sorafenib docked to 1UWJ						Rank	PLX4720 docked to 3C4C					
	D^3DOCKxb		Autodock		Glide			D^3DOCKxb		Autodock		Glide	
	Binding Score (kcal/mol)	Rmsd(Å)	Binding Score (kcal/mol)	Rmsd(Å)	Binding Score (kcal/mol)	Rmsd(Å)		Binding Score (kcal/mol)	Rmsd(Å)	Binding Score (kcal/mol)	Rmsd(Å)	Binding Score (kcal/mol)	Rmsd(Å)
1	-11.42	1.15	-11.18	1.16	-12.08	0.85	1	-10.40	8.04	-10.20	0.71	-11.45	0.48
2	-11.42	1.21	-11.17	1.13	-11.42	0.97	2	-10.26	0.32	-10.19	0.71	-11.23	0.56
3	-11.41	1.08	-11.17	1.16	-11.19	0.61	3	-10.25	0.35	-10.19	0.84	-11.06	0.62
4	-11.41	1.09	-11.17	1.09	-11.09	2.16	4	-10.24	0.34	-10.19	0.70	-10.89	0.62
5	-11.40	1.10	-11.16	0.92	-10.99	1.00	5	-10.24	0.34	-10.18	0.32	-10.86	0.66
6	-11.40	1.15	-11.16	1.16	-10.91	2.45	6	-10.24	0.33	-10.18	0.37	-10.80	1.07
7	-11.39	1.08	-11.16	0.89	-10.29	1.34	7	-10.23	0.31	-10.18	0.69	-10.79	0.62
8	-11.38	1.07	-11.15	1.19	-10.17	0.84	8	-10.23	0.34	-10.17	0.71	-10.76	0.96
9	-11.37	1.13	-11.15	1.13	-9.94	2.32	9	-10.23	0.40	-10.17	0.41	-10.70	0.94
10	-11.37	1.13	-11.15	0.87	-9.91	1.41	10	-10.22	0.38	-10.17	0.78	-10.53	0.93
11	-11.37	1.08	-11.15	0.91	-9.65	1.12	11	-10.22	0.36	-10.16	0.39	-9.93	1.04
12	-11.36	1.20	-11.15	0.87	-9.45	2.43	12	-10.22	0.35	-10.16	0.75	-9.62	2.08
13	-11.36	1.06	-11.15	0.91	-9.19	0.89	13	-10.22	0.39	-10.16	0.76	-9.42	2.08
14	-11.36	1.07	-11.15	0.87	-8.61	2.41	14	-10.21	0.96	-10.16	0.43	-9.07	1.09
15	-11.36	1.14	-11.15	0.90	-7.72	2.69	15	-10.21	0.36	-10.16	0.67	-9.06	1.29
16	-11.35	1.11	-11.14	0.93	-6.75	8.01	16	-10.21	0.36	-10.15	0.40	-8.38	2.16
17	-11.35	1.12	-11.14	1.10	-6.75	7.76	17	-10.21	0.37	-10.15	0.80	-8.20	2.15
18	-11.35	1.05	-11.14	0.90	-6.42	7.77	18	-10.21	0.40	-10.14	0.40	-8.15	2.48
19	-11.35	1.07	-11.14	1.16	-6.25	7.85	19	-10.21	0.45	-10.14	0.87	-8.12	2.66
20	-11.35	1.07	-11.14	1.10	-	-	20	-10.20	0.40	-10.14	0.47	-8.08	2.43

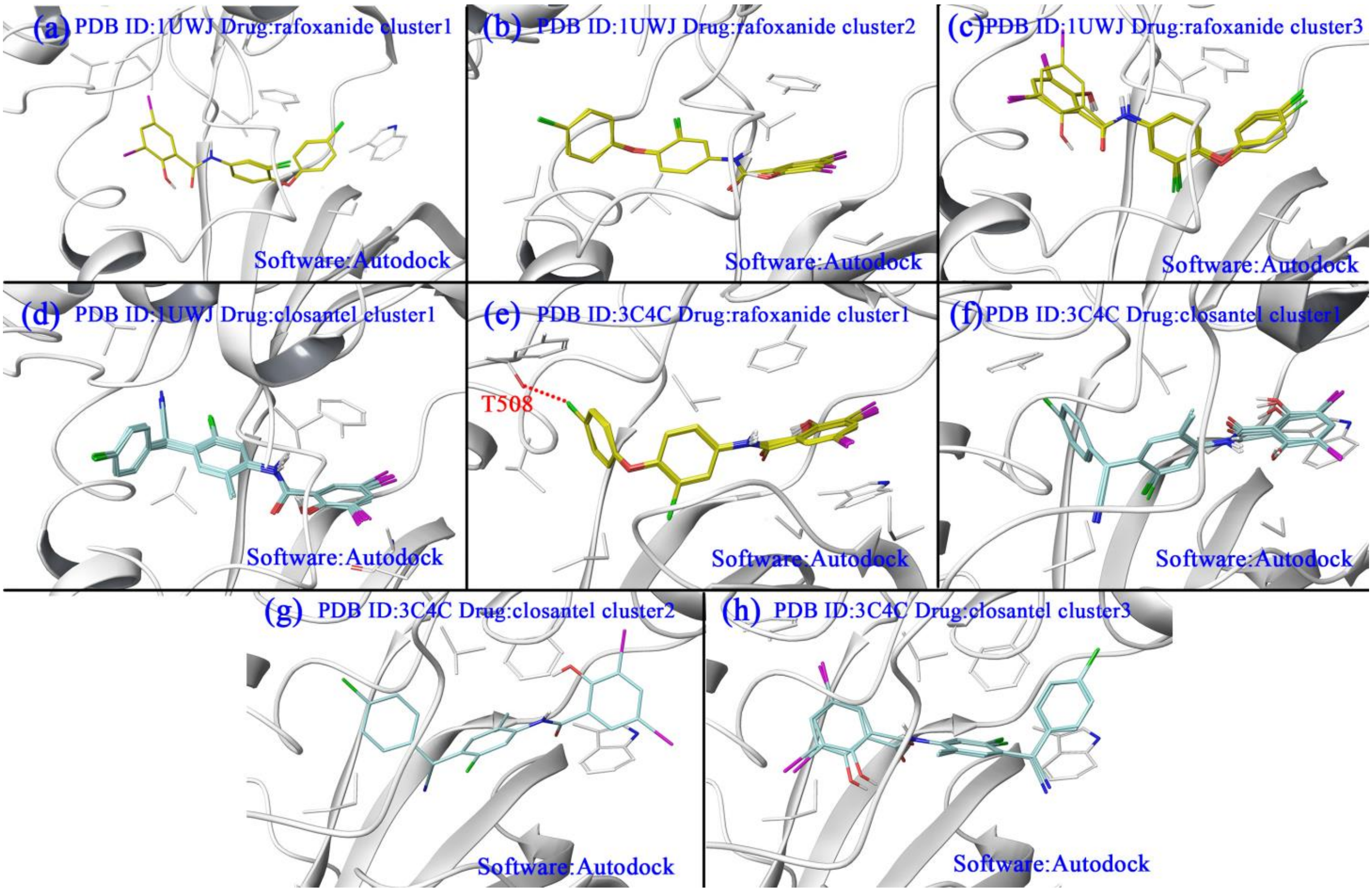

Supplementary Figure S4. Rafoxanide and closantel docked in 1UWJ and 3C4C by Autodock. Top10 conformations were clustered in each case. Halogen bond interactions were labeled according to the best scored conformation in each cluster.

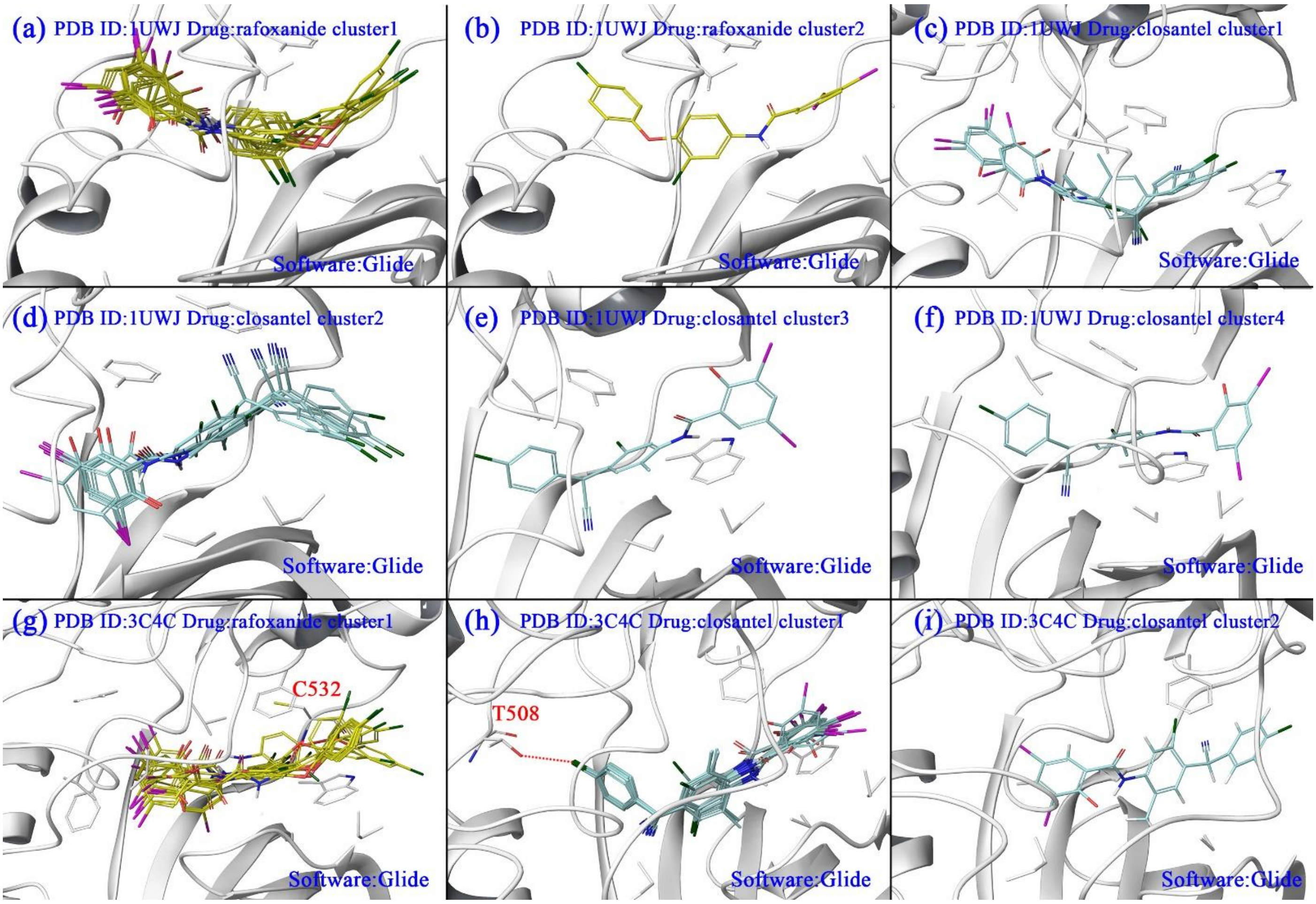

Supplementary Figure S5. Rafoxanide and closantel docked in 1UWJ and 3C4C by Glide. Top10 conformations were clustered in each case. Halogen bond interactions were labeled according to the best scored conformation in each cluster.

Supplementary Table S3. Cluster information of rafoxanide and closantel docked in 1UWJ and 3C4C by Autodock. Binding scores and the rmsd towards the best scored conformation in cluster were listed.

Software	PDB ID	Drug	Cluster	Binding Score Rank	Binding Score (kcal/mol)	Cluster RMSD(Å)
Autodock	1UWJ	rafoxanide	1	1	-12.00	0.00
Autodock	1UWJ	rafoxanide	1	5	-11.99	0.12
Autodock	1UWJ	rafoxanide	2	3	-12.00	0.00
Autodock	1UWJ	rafoxanide	2	4	-12.00	0.22
Autodock	1UWJ	rafoxanide	3	2	-12.00	0.00
Autodock	1UWJ	rafoxanide	3	6	-11.98	2.13
Autodock	1UWJ	rafoxanide	3	7	-11.97	2.13
Autodock	1UWJ	rafoxanide	3	8	-11.96	2.12
Autodock	1UWJ	rafoxanide	3	9	-11.95	2.12
Autodock	1UWJ	rafoxanide	3	10	-11.94	2.12
Autodock	1UWJ	closantel	1	1	-12.53	0.00
Autodock	1UWJ	closantel	1	2	-12.50	0.19
Autodock	1UWJ	closantel	1	3	-12.47	0.20
Autodock	1UWJ	closantel	1	4	-12.37	0.37
Autodock	1UWJ	closantel	1	5	-12.36	0.25
Autodock	1UWJ	closantel	1	6	-12.15	0.16
Autodock	1UWJ	closantel	1	7	-12.15	0.43
Autodock	1UWJ	closantel	1	8	-12.13	0.29
Autodock	1UWJ	closantel	1	9	-12.03	2.57
Autodock	1UWJ	closantel	1	10	-11.66	2.04
Autodock	3C4C	rafoxanide	1	1	-10.72	0.00
Autodock	3C4C	rafoxanide	1	2	-10.71	0.13
Autodock	3C4C	rafoxanide	1	3	-10.71	0.17
Autodock	3C4C	rafoxanide	1	4	-10.70	0.07
Autodock	3C4C	rafoxanide	1	5	-10.70	0.23
Autodock	3C4C	rafoxanide	1	6	-10.70	0.06
Autodock	3C4C	rafoxanide	1	7	-10.69	0.13
Autodock	3C4C	rafoxanide	1	8	-10.69	0.09
Autodock	3C4C	rafoxanide	1	9	-10.69	0.25
Autodock	3C4C	rafoxanide	1	10	-10.69	0.23
Autodock	3C4C	closantel	1	1	-11.25	0.00
Autodock	3C4C	closantel	1	2	-11.10	0.19
Autodock	3C4C	closantel	1	3	-10.87	0.25
Autodock	3C4C	closantel	1	5	-10.76	2.02
Autodock	3C4C	closantel	1	6	-10.75	2.02
Autodock	3C4C	closantel	1	7	-10.72	2.02
Autodock	3C4C	closantel	2	4	-10.81	0.00
Autodock	3C4C	closantel	3	8	-10.66	0.00
Autodock	3C4C	closantel	3	9	-10.66	0.36
Autodock	3C4C	closantel	3	10	-10.65	0.03

Supplementary Table S4. Cluster information of rafoxanide and closantel docked in 1UWJ and 3C4C by Glide. Binding scores and the rmsd towards the best scored conformation in cluster were listed.

Software	PDB ID	Drug	Cluster	Binding Score Rank	Binding Score (kcal/mol)	Cluster RMSD(Å)
Glide	1UWJ	rafoxanide	1	1	-8.61	0.00
				2	-8.58	2.11
				3	-8.48	1.36
				4	-8.48	2.28
				5	-8.45	2.48
				6	-8.27	2.52
				7	-8.14	2.25
				9	-7.99	1.60
				10	-7.75	2.92
			2	8	-8.00	0.00
		closantel	1	1	-8.73	0.00
				2	-8.44	3.24
				4	-7.63	3.77
			2	5	-7.33	0.00
				6	-7.28	2.08
				7	-6.94	2.03
				9	-6.48	2.17
				10	-6.33	2.52
			3	3	-7.72	0.00
			4	8	-6.93	0.00
	3C4C	rafoxanide	1	1	-7.21	0.00
				2	-7.15	0.85
				3	-7.08	1.37
				4	-6.71	2.21
				5	-6.64	2.46
				6	-6.58	2.44
				7	-6.57	2.12
				8	-6.56	1.80
				9	-6.56	1.42
				10	-6.49	2.74
		closantel	1	1	-7.66	0.00
				3	-7.53	2.01
				4	-7.50	2.03
				5	-7.49	1.12
				6	-7.35	1.98
				7	-7.29	2.67
				8	-7.28	2.09
				9	-7.06	1.27
				10	-7.06	2.68
			2	2	-7.58	0.00
